# Toward Enhancing Wearability and Fashion of Wearable Supercapacitor with Modified Polyurethane Artificial Leather Electrolyte

**DOI:** 10.1007/s40820-018-0191-7

**Published:** 2018-02-16

**Authors:** Yan Huang, Zijie Tang, Zhuoxin Liu, Jun Wei, Hong Hu, Chunyi Zhi

**Affiliations:** 1 State Key Laboratory of Advanced Welding and Joining, Harbin Institute of Technology (Shenzhen), Shenzhen, 518055 People’s Republic of China; 2 Center of Flexible and Printable Electronics, Harbin Institute of Technology (Shenzhen), Shenzhen, 518055 People’s Republic of China; 3 Department of Materials Science and Science, City University of Hong Kong, 83 Tat Chee Avenue, Hong Kong, People’s Republic of China; 4 Singapore Institute of Manufacturing Technology, 73 Nanyang Drive, Singapore, Singapore; 5 Institute of Textiles and Clothing, The Hong Kong Polytechnic University, 11 Hong Chong Road, Hong Kong, People’s Republic of China

**Keywords:** Artificial leather, Neutral electrolyte, Wearable supercapacitor, Fluorescence

## Abstract

Inspired by the sophisticated artificial leather garment industry and toward enhancing wearability of energy storage devices, we demonstrate a polyurethane artificial leather supercapacitor with large sheet electrodes embedded in the leather layer simultaneously working as a polyelectrolyte. This design totally reserves textiles underneath and thus addresses the well-known challenge of wearing comfortability. It provides a revolutionary configuration of wearable supercapacitors: the artificial leather on garment is also a supercapacitor. Unlike the polyvinyl alcohol-based acidic electrolytes, which are widely used, sodium chloride is used to modify the intrinsically fluorescent polyurethane leather for ionic transportation, which has no harm to human. The fluorescent leather supercapacitor is easily transferrable from any arbitrary substrates to form various patterns, enabling multifunctionalities of practical wearability, fashion, and energy storage.

**Electronic supplementary material:**

The online version of this article (10.1007/s40820-018-0191-7) contains supplementary material, which is available to authorized users.

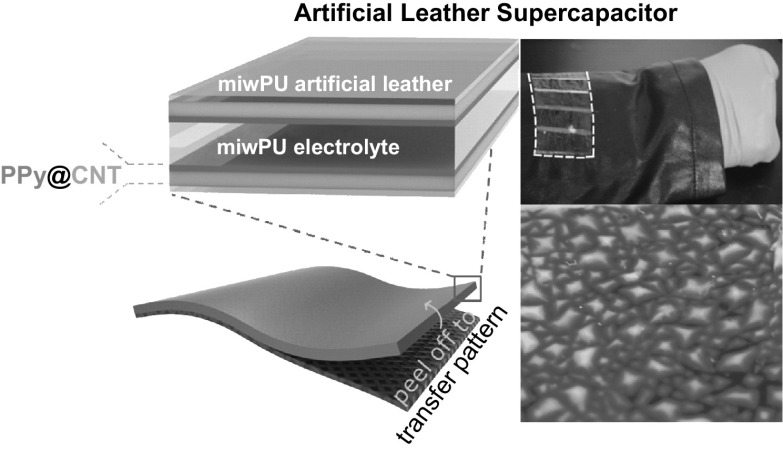

## Highlights

- Practically wearable, easily transferrable, and fluorescent artificial leather supercapacitor was fabricated by combining energy storage technology with leather garment industry, solving the intrinsic problem of wearing comfortability in conventional yarn and textile supercapacitors.
- Polyurethane as an important artificial leather is modified to be ion conductive by the incorporation of ionic groups and non-hazardous sodium chloride. The modified polyurethane artificial leather serves as a polyelectrolyte simultaneously.
- The intrinsically fluorescent artificial leather supercapacitor is easily transferrable from any arbitrary substrates to form various patterns, enabling multifunctionalities of practical wearability, fashion, and energy storage.

## Introduction

Wearable energy storage devices are a critical element in personalized wearable electronics [1–4]. There is no doubt that textiles are most wearable due to their good comfortability and texture etc., which eventually arise from the micron size of polymeric filaments (< 5 µm) [1, 5]. When a bunch of such thin filaments are twisted to yarns and then further weaved/knitted to textiles, the softness as well as the concomitant comfortability and texture are well maintained. Although many yarn and textile supercapacitors [6–11] have been developed and become one mainstream of wearable devices [12–16], this conventional form loses the comfortability and texture due to the incorporation of polyelectrolyte. As the thickness of polyelectrolytes (far larger than 100 µm) is much higher than the aforementioned comforting filament size (< 5 µm), these yarn and textile supercapacitors reported so far give rise to practical wearability challenges. Thus, the widely used form of yarn and textile supercapacitors fundamentally attributes to the limitation of insufficient wearability, which impose a considerable requirement on reforming wearable supercapacitors.

The reformed wearable supercapacitor should maximize wearability. This requires maximum reservation of traditional textiles. Inspired by the configuration of leather garment, we propose an artificial leather supercapacitor as an alternative approach. By the design of supercapacitor in the layer of artificial leather on a leather garment, traditional textiles underneath can be totally reserved. This achieves the same comfortability and wearability as of common leather garment. This makes the artificial leather supercapacitor an excellent reform for wearable supercapacitors.

Notably, compared with polyvinyl alcohol (PVA), polyurethane (PU) is widely used as an artificial leather. Nevertheless, pristine PU is not ion conductive and cannot work as an electrolyte. In this paper, PU is modified to be ion conductive by the incorporation of ionic groups in aqueous solvent, and non-hazardous sodium chloride (NaCl) is used to further improve the ionic transportation, avoiding the potential harm of acid to human. There are no functional groups on the backbone of the modified PU that restrain free sodium ions, thus providing decent ion conductivity. Moreover, PU is easily transferrable from arbitrary substrates and intrinsically fluorescent. These are not exhibited by the widely used PVA-based electrolytes and provide potential fashion garment application. As a proof-of-concept study, a supercapacitor sleeve is fabricated by using large carbon nanotube (CNT) sheet electrodes deposited with polypyrrole (PPy) and the modified PU as both electrolyte and artificial leather, which emits near-blue fluorescence and powers a light-emitting diode.

## Experimental

### Modification of Water-Based PU and Fabrication of the Artificial Leather Supercapacitor

Carboxyl groups were introduced into the water-based PU (wPU), and various NaCl (0.025–0.25 M) and dyes (10 µm) were added into the resultant ionic wPU (iwPU) dispersion under vigorous stirring for 0.5 h. The as-mixed dispersion was dried at room temperature to form miwPU films. Then, CNT sheets were electrodeposited with PPy up to 1.2 mg at 0.8 V versus Ag/AgCl up to 5 min at 0 °C, in an electrolyte solution (30 mL) containing 0.1 M p-toluenesulfonic acid, 0.3 M sodium toluenesulfonate, and 15 µL pyrrole monomer. Besides serving as the suppliers of p-toluenesulfonate, they can stabilize the pH of the solution, which is a key factor in the PPy electropolymerization. Pyrrole monomers were purified by distilling before electrodeposition. Two PPy@CNT sheets were on each side of the miwPU polyelectrolyte film. Finally, two miwPU artificial leathers were deposited on these two sheets.

### Electrochemical Characterization

CV and GCD of supercapacitor devices were tested by a potentiostat (CHI 760E) at room temperature. The capacitance of the single electrode (*C*_m_) was calculated from GCD and CV curves according to Eqs. 1 and 2, respectively:

1 $$C_{\text{m}} = \frac{2It}{Um}$$

2 $$C_{m} = \frac{1}{Uvm}\mathop \smallint \limits_{{U_{ - } }}^{{U_{ + } }} i\left( U \right){\text{d}}U$$

where *I* is the discharge current of GCD, *t* is the discharge time of GCD, *U* is the voltage window (*U* = *U*_+_ − *U*_−_), *m* is the mass of PPy electrode, $v$ is the scan rate of CV, and *i*(U) is the current of CV.

## Results and Discussion

### Modification and Physicochemical Properties of PU Artificial Leather as a Polyelectrolyte

**Fig. 1:**
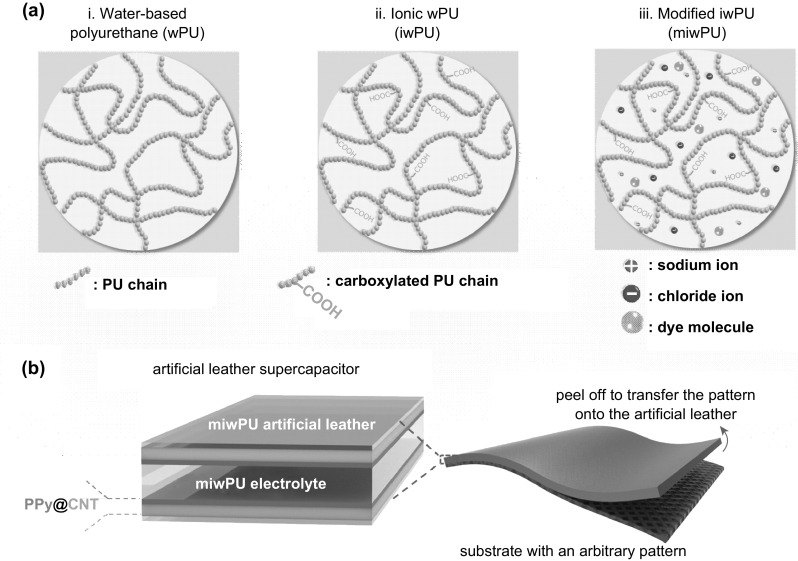
Schematics of PU artificial leather modification to serve as a polyelectrolyte of an artificial leather supercapacitor. **a** From wPU to miwPU: (i) wPU without free ions, (ii) iwPU with ionic groups grafted on polymer chains, (iii) miwPU with the presence of NaCl and dyes. **b** The artificial leather supercapacitor comprising the miwPU electrolyte, PPy@CNT sheet electrodes, and miwPU artificial leather with patterns transferred from an arbitrary substrate

As an ideal substitute for natural leather, PU artificial leather has been substantially applied in the garment industry. wPU has gradually replaced that of the organic solvent-based and becomes an important direction for industrial development. As illustrated in Fig. 1a_i_, PU chains are well dispersed in the water. When ionic groups such as carboxyls are grafted on PU chains, it becomes iwPU (Fig. 1a_ii_). During the pre-step polymerization of wPU monomers, carboxyl-contained chain extenders are added and then grafted on PU chains after polymerization. Its ionic conductivity is enhanced due to the incorporation of ions. Non-hazardous strong electrolyte NaCl as well as a few dye molecules (10 µM) is added to further modify the iwPU (miwPU) in order to remarkably increase the ion concentration and to display various colors for serving as both a harmless electrolyte and a colored artificial leather simultaneously (Fig. 1a_iii_, and Fig. S1). Therefore, the miwPU film is used as the electrolyte and the artificial leather. CNT sheets and PPy thin films electrodeposited are employed as current collectors and active materials, respectively (Fig. 1b). Besides capacitance contribution, the intrinsically flexible and stable PPy serves as a good stress buffer which is a desirable feature of wearable devices because they always have to experience various deformations [17–21]. The whole supercapacitor is imbedded in the miwPU film to form an artificial leather supercapacitor. Arbitrary patterns are easily transferred onto the miwPU artificial leather supercapacitor by peeling off from arbitrary substrates. Such arbitrary pattern transferring is unique in most publications on wearable supercapacitors due to the use of the artificial leather.

**Fig. 2:**
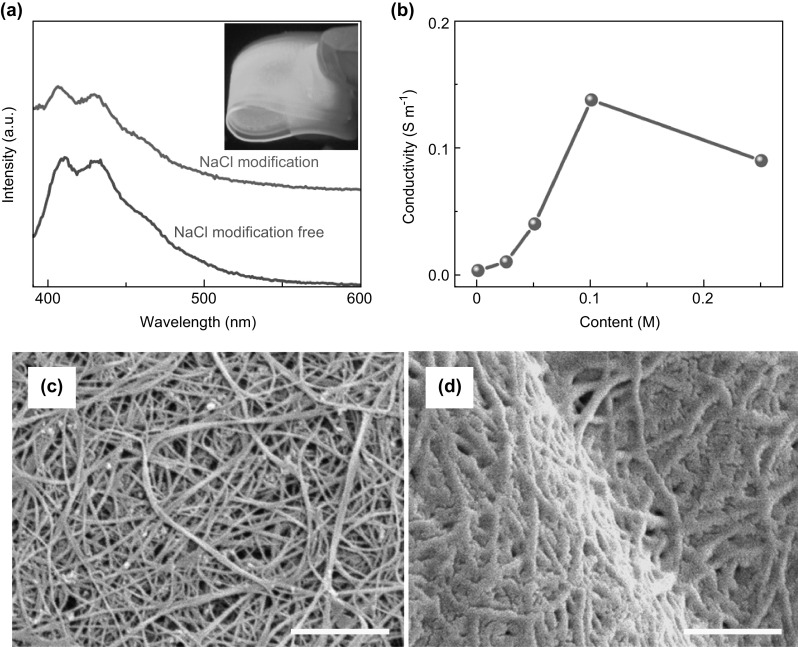
**a** Fluorescence emission spectra of miwPU films with and without NaCl modification when excited at 365 nm (Inset is a photograph of the miwPU film under the excitation). **b** Ionic conductivities at various contents of NaCl in the miwPU film. **c** An SEM picture of CNT sheets (Scale bar is 1 µm). **d** An SEM picture of the CNT sheets on which PPy is electrodeposited (Scale bar is 1 µm)

As demonstrated in Fig. 2a, two main peaks at around 409 and 430 nm are observed in the emission spectra, which correspond to the blue fluorescence. The intrinsic fluorescence behavior of iwPU is not changed by the addition of NaCl, validating the feasibility as multifunctional miwPU. The fluorescence plays an important role in endowing the artificial leather supercapacitor an element of fashion. Raman spectra and mechanical performances are also unaffected by NaCl (Fig. S2), indicating an absence of bond interactions and the existence of free ions. A morphology observation of cryo-dried samples using scanning electron microscopy (SEM) demonstrates that a porous microstructure appears both in iwPU and miwPU with the pore size around 100 µm (Fig. S3). The absence of bond interactions and the existence of free ions result in decent ionic conductivity of miwPU (Fig. 2b). The ionic conductivity remarkably changes with the content of NaCl. With the increase in the NaCl content, the conductivity substantially increases with the concentration and then decreases, appearing the highest value. This suggests that the concentration affects the conductivity from two opposite directions. For very dilute concentration, when it increases, the increase of ion amounts plays a dominant role and thus the conductivity increases. When the concentration increases to 0.25 M, the ionic interaction becomes a dominant role and therefore the conductivity decreases. Together with the miwPU as the polyelectrolyte, PPy@CNT sheet electrodes are utilized to fabricate the wearable, transferrable, and fluorescent supercapacitor. Raman spectra confirmed the species of CNT and PPy (Fig. S4). Figure 2c, d shows that CNT sheets are made of interweaved nanowires, and the electrodeposited PPy is a uniform thin film on CNT sheets. It is noteworthy that there is no crack on the intentionally folded electrodes due to their great flexibility.

### Electrochemical Characterization

**Fig. 3:**
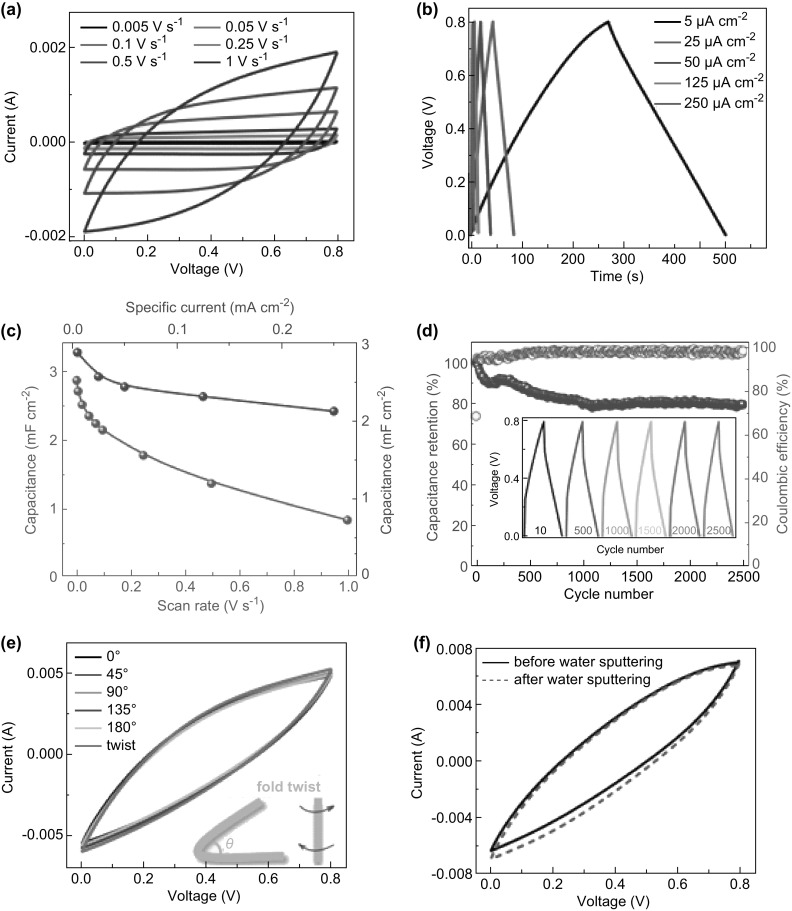
**a** CV profiles at scan rates ranging from 0.005 to 1 V s^−1^. **b** GCD profiles at current densities ranging from 5 to 250 µA cm^−2^. **c** Areal capacitances calculated according to CV (red) and GCD (blue) profiles of the PPy@CNT sheet electrode using the miwPU polyelectrolyte. **d** Charge/discharge cycling stability (Inset is GCD curves at various cycle numbers). **e** CV profiles of the supercapacitor undergoing consecutive deformations (scan rate: 0.05 V s^−1^). **f** CV profiles of the waterproof spray-sprinkled supercapacitor prior to and after water sputtering (scan rate: 0.1 V s^−1^)

Electrochemical performances are systematically studied on the miwPU polyelectrolyte in the supercapacitor as aforedescribed. Figure 3a, b shows a series of cyclic voltammetry (CV) and galvanostatic charge/discharge (GCD) profiles. At scan rates up to 0.25 V s^−1^, CVs display a rectangular shape. At higher scan rates of 0.5 and 1 V s^−1^, CVs start to display some features of a resistor. This mainly arises from higher ionic transfer resistance due to the diffusion limitation [20]. For all current densities studied here, the supercapacitor retains the shape of almost isosceles triangles. Although both CNT and PPy contribute to the capacitance, it is clearly seen that the contribution from PPy is much higher (~ 11 times) than that of CNT (Fig. S5), validating the use of PPy as the electrodes. Figure 3c shows the areal capacitances calculated from the CV and GCD profiles above. Considered the large area of an artificial leather garment, the total capacitance could be enhanced vastly. Mass capacitances of PPy up to 105 F g^−1^ are shown in Fig. S6, which are comparable to other reports [22–27]. By contrast to capacitances obtained from acidic polyelectrolytes, these results are lower mainly due to the inferior ionic conductivity of NaCl than that of acid [17, 20, 21]. Consistent with the ionic conductivity trend with the NaCl content, the capacitance of the miwPU polyelectrolyte initially increases with the concentration and then decreases (Fig. S7). In addition to NaCl concentration, the water content also greatly affects electrochemical performances (Fig. S8). The solvent water exists in the gel, forming a free-standing polyelectrolyte without any fluidity and therefore avoiding the risk of leakage. The cycling stability test shows that the supercapacitor has a capacitance reservation of 80% after over 2500 cycles with Coulombic efficiency ~ 100% during all these cycles (Fig. 3d), which are better than PPy-based supercapacitors reported [23]. The initial degradation should be attributed to the loss of the water solvent. All GCD profiles are similar in the whole cycling test and even almost overlay since the 1000th cycle, indicating unchanged electrochemical dynamic processes. Performance comparisons are summarized in Table S1.

Deformation stability is definitely required for practical wearing applications. Our supercapacitor experiences a series of deformation test, such as being folded at 0°, 45°, 90°, 135°, 180°, and twisted. All CV curves overlap almost completely during the whole deformation process (Fig. 3e), revealing the excellent device flexibility required for wearable electronics due to the good mechanical property of the PU gel [28] (Fig. S2). Moreover, another important feature for practical wearable applications is being impervious to water. By sprinkling waterproof spray onto the miwPU artificial leather supercapacitor, the CV curves are almost identical before and after water sputtering (Fig. 3f), suggesting a potential solution to all wearable devices with water-compatible polyelectrolytes.

### Wear the Fluorescent miwPU Artificial Leather Supercapacitor Sleeve

**Fig. 4:**
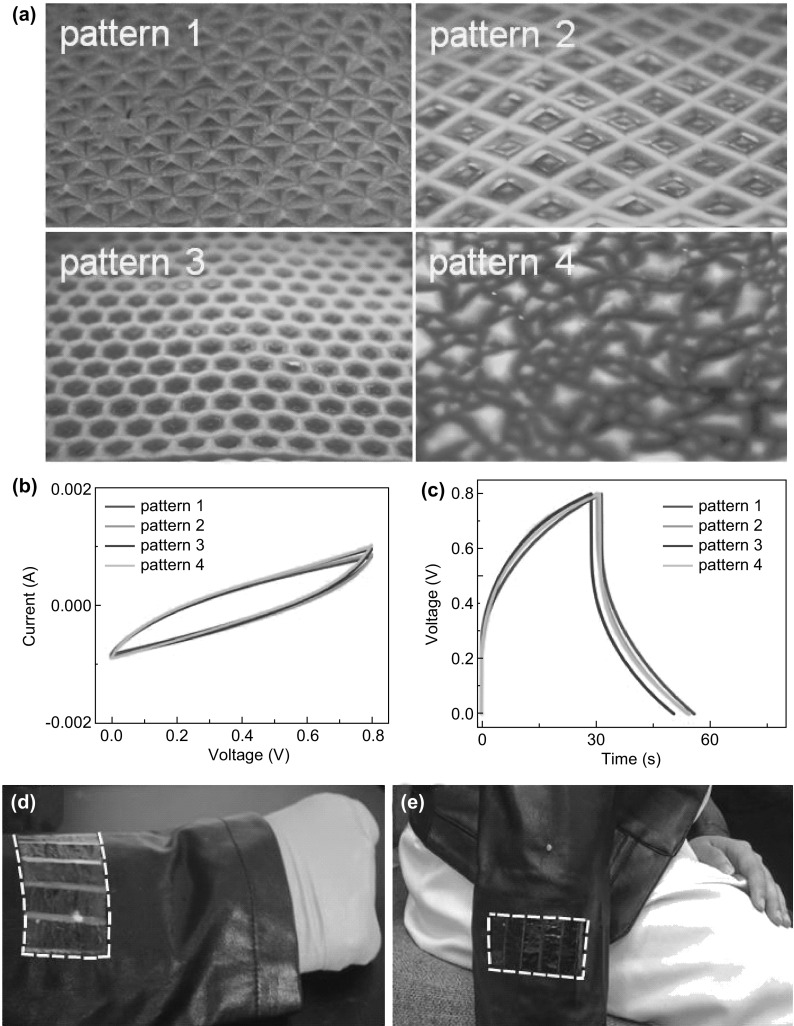
**a** Fluorescent miwPU artificial leathers with various patterns. **b** CV curves of patterned miwPU artificial leather supercapacitors. **c** GCD curves of patterned miwPU artificial leather supercapacitors. **d** The fluorescent supercapacitor sleeve under excitation. **e** The supercapacitor sleeve powering a light-emitting diode

The miwPU artificial leather supercapacitor demonstrates an excellent pattern diversity (Fig. 4a). Arbitrary patterns can be easily transferred onto the leather by just peeling off from any patterned substrates. All patterned leathers exhibit the fluorescence effect with slight differences, which is likely caused by their color differences due to the use of different dyes in the miwPU as shown in Fig. S1. The electrochemical performances are not affected by either the patterns or the colors (Fig. 4b, c and Fig. S9), validating the integration of supercapacitor and artificial leather. Notably, the IR drop in the discharging curve is relatively large due to the low water solvent content in the gel electrolyte.

Two to four supercapacitors are assembled both in parallel (Fig. S10a, b) and in series (Fig. S10c, d) for practical applications. The charge/discharge time and thus the overall capacitance increase linearly with the number of supercapacitors in parallel. Similarly, the overall capacitance linearly decreases with the reciprocal of the number of supercapacitors in series. The combination of good flexibility and scalability of supercapacitors validates the wearable application. We fabricate a supercapacitor sleeve by using in-series and in-parallel assemblies, which displays the fluorescence effect (Fig. 4d) and powers a light-emitting diode (Fig. 4e).

## Conclusions

In summary, polyurethane artificial leather is modified with carboxyl groups and non-hazardous sodium chloride to serve as a polyelectrolyte and meanwhile maintains the intrinsic property of fluorescence effect. The artificial leather supercapacitor using sheet electrodes exhibits excellent flexibility, pattern diversity, scalability, and high compatibility with artificial leather industrial processing techniques. As a demonstration, a fluorescent supercapacitor sleeve is fabricated by using these supercapacitors to power a light-emitting diode, realizing the energy storage, fluorescence capability, and wearability. Inheriting from the great wearability of artificial leather garment, the artificial leather supercapacitor has no problem of the practical comforting wearability of textiles, thus providing a reform of the mainstream yarn/textile-based supercapacitors and creating considerable potential for more practical applications of wearable electronics.

## Electronic supplementary material

Below is the link to the electronic supplementary material.

Supplementary material 1 (PDF 558 kb)
