# Supplementary material for: Toward Enhancing Wearability and Fashion of Wearable Supercapacitor with Modified Polyurethane Artificial Leather Electrolyte
Source: Nanomicro Lett. 2018 Feb 16;10(3):38. doi: 10.1007/s40820-018-0191-7 (PMC6199084; doi:10.1007/s40820-018-0191-7)
Supplement: Supplementary file 1 — Supplementary material 1 (PDF 558 kb) [file 40820_2018_191_MOESM1_ESM.pdf]

Supporting Information

Toward enhancing wearability and fashion of wearable supercapacitor with modified polyurethane artificial leather electrolyte

Yan Huang,[1,2] Zijie Tang,[3] Zhuoxin Liu,[3] Jun Wei,[4] Hong Hu,[5] and Chunyi Zhi[3]*

[1]State Key Laboratory of Advanced Welding and Joining, Harbin Institute of Technology (Shenzhen), Shenzhen 518055, China.

[2]Center of Flexible and Printable Electronics, Harbin Institute of Technology (Shenzhen), Shenzhen 518055, China.

[3]Department of Materials Science and Science, City University of Hong Kong, 83 Tat Chee Avenue, Hong Kong, China.

[4]Singapore Institute of Manufacturing Technology, 73 Nanyang Drive, Singapore.

[5]Institute of Textiles and Clothing, The Hong Kong Polytechnic University, 11 Hong Chong Road, Hong Kong, China.

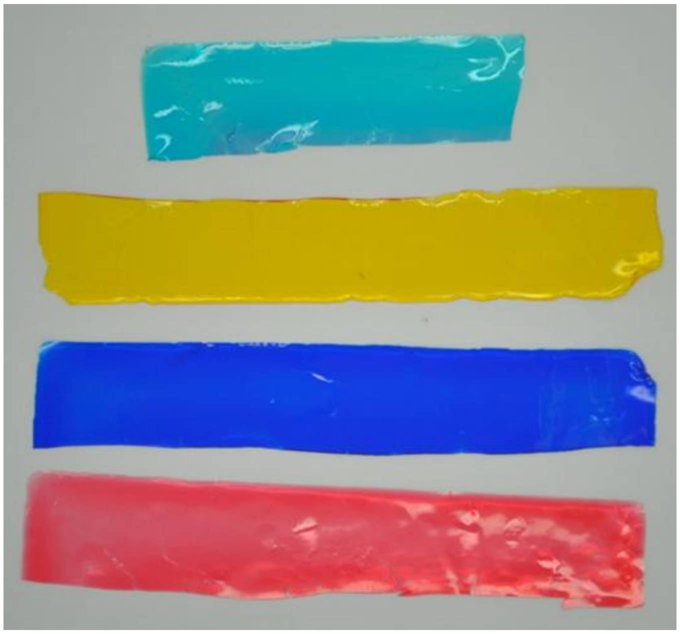

Figure S1. Photograph of miwPU showing various colors.

With the use of different dyes, miwPU artificial leathers display multiple colors. This demonstrates its potential for fashion show.

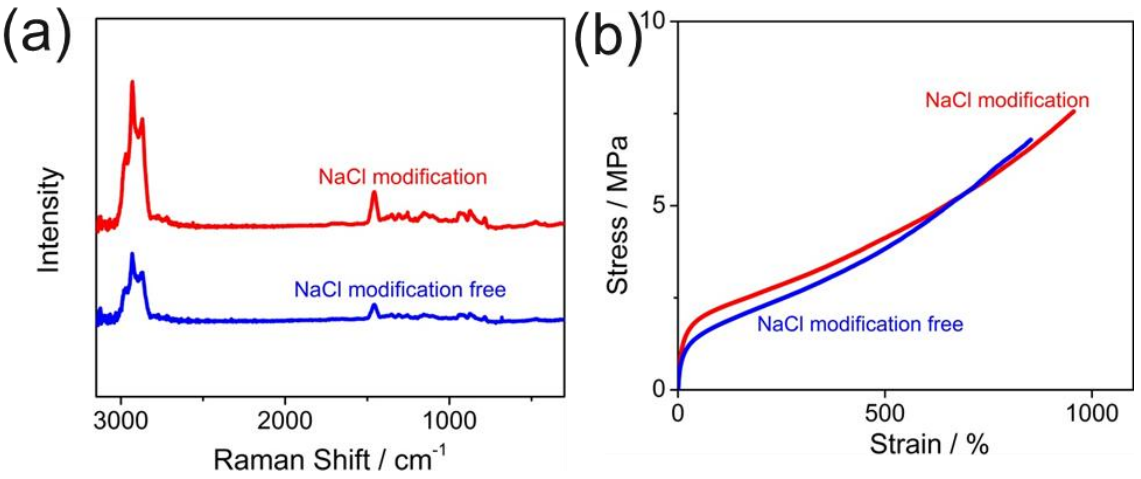

Figure S2. Raman spectra (a), and mechanical performances (b) of iwPU with vs. without NaCl modification.

Both Raman spectra show the characteristic peak at 1459 cm^{-1} which corresponds to the aliphatic CH_2 stretch. Furthermore, two peaks at 2931cm^{-1} and 2868 cm^{-1} are assigned to the C–H stretching vibration of the aliphatic chain and protonated amine stretched deformation vibration.[S1] The almost identical spectra indicates an absence of bond interactions and the existence of free ions.

The addition of NaCl also has negligible effect on the mechanical properties. The iwPU can sustain over 800% strain, and has tensile strength over 7 MPa. The similar mechanical properties could be attributed to the small amount and/or ion size of NaCl added.

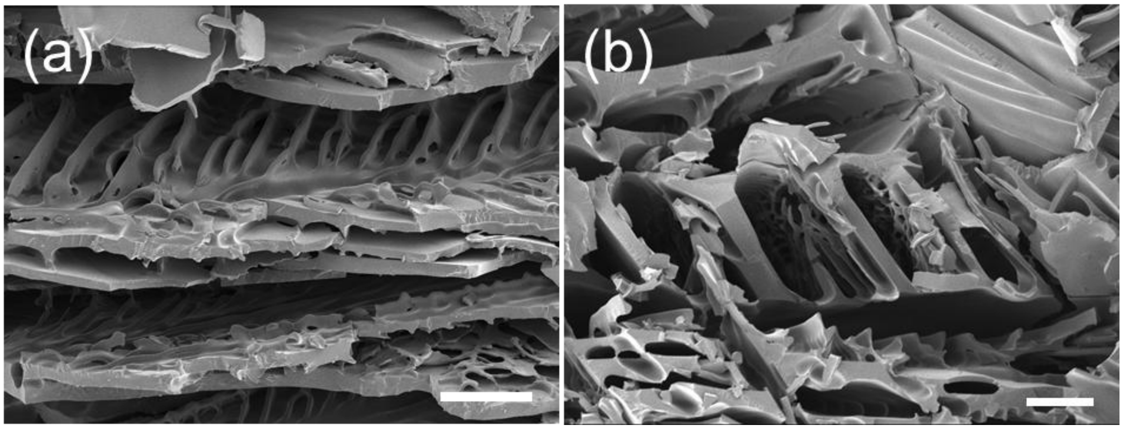

Figure S3. SEM images of the iwPU (a), and miwPU (b) after freeze drying. Scale bar, 100 μm.

Both iwPU and miwPU demonstrate a porous microstructure with the pore size around 100 μm. The iwPU shows the front view, and miwPU mainly shows the top view of the porous structure.

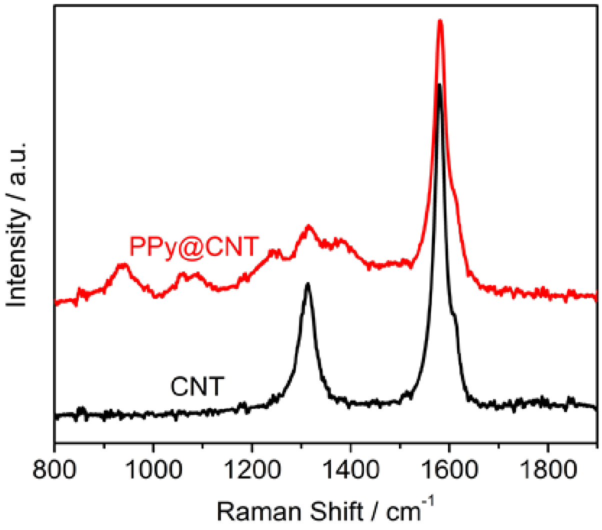

Figure S4. Raman spectra of CNT and PPy.

Two typical peaks of CNT at 1312 cm^{-1} and 1581 cm^{-1} are observed, which correspond to the D band (from sp^2 at defects of carbon hexagonal symmetry) and the G band (in-plane vibration of carbon atoms with an sp^2 bond), respectively[S2]. The Raman spectrum confirms the species of PPy. The peak at around 937 cm^{-1} corresponds to ring deformation. The band at 987 cm^{-1} is attributed to ring deformation associated with dictation. The peaks at 1059 cm^{-1} and 1089 cm^{-1} correspond to the symmetrical C–H in-plane bending and N–H in-plane deformation. The peaks at 1248 cm^{-1} and 1312 cm^{-1} are attributed to the antisymmetrical C–H in-plane bending and antisymmetrical in-ring C–N stretching, respectively. C–C and C–N stretching are reflected at both 1383 cm^{-1} and 1506 cm^{-1}. Beside the G band of CNT, the peak at 1580 cm^{-1} is an overlap of C–C in-ring and C–C inter-ring stretching resulting from radical cations and dictation[S3].

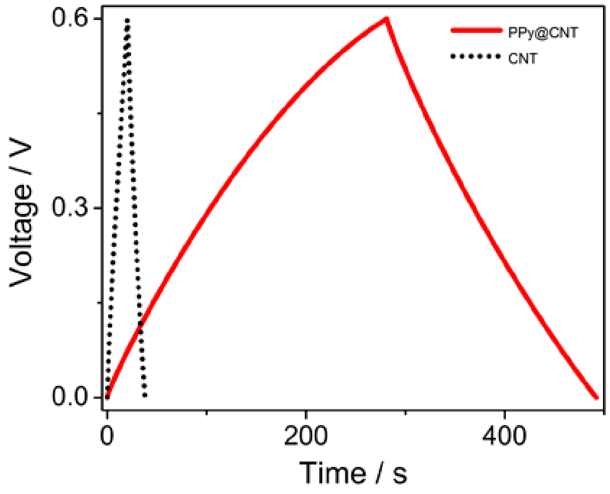

Figure S5. GCD curves of the PPy@CNT and pure CNT electrodes.

CNT and PPy both contribute to the capacitance. However, the contribution from PPy is much higher (~11 times) than CNT.

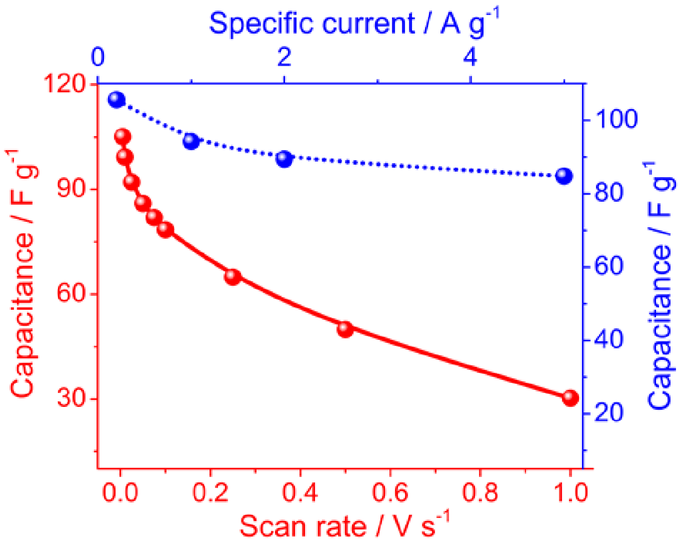

Figure S6. Mass capacitances of PPy calculated from CV (red) and GCD (blue) curves of the PPy@CNT sheet electrode using the miwPU polyelectrolyte.

The mass capacitances of PPy are comparable to those tested in liquid electrolytes.

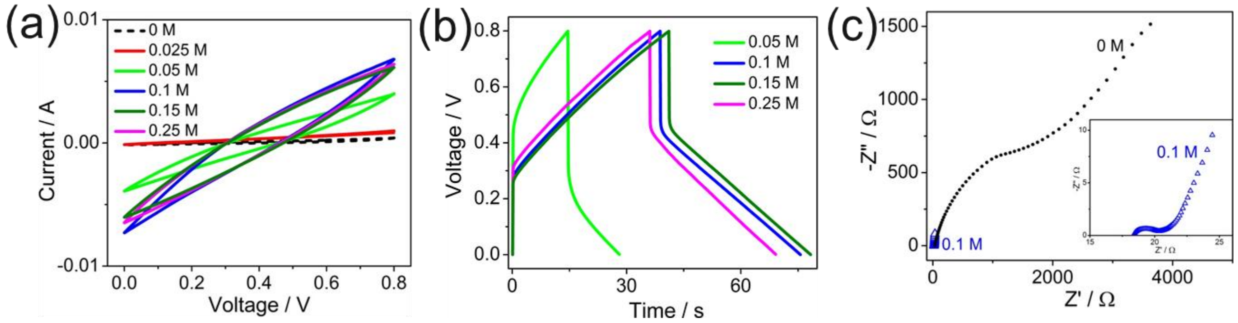

Figure S7. CV (a), GCD curves (b), and Nyquist plots (c) of the miwPU polyelectrolyte with various concentrations of NaCl in the miwPU dispersion.

Consistent with the results that ionic conductivity is affected by NaCl content in the miwPU polyelectrolyte, the CV and GCD curves are also remarkably different at various NaCl contents. Both the CV and GCD curves first get larger and then smaller with the increased NaCl content. The varied capacitances are determined by the change of ionic conductivity. Compared with the electrolyte without NaCl, the supercapacitor with the presence of 0.1 M NaCl exhibits much smaller systematic resistance (the intercept at the Z`-axis) and overall impedance (the end point in the Nyquist plot). The sufficiently available ions favor ion transportation in the electrolyte and at the electrolyte/electrode interface, thereby reducing the resistance and increasing the capacitance.

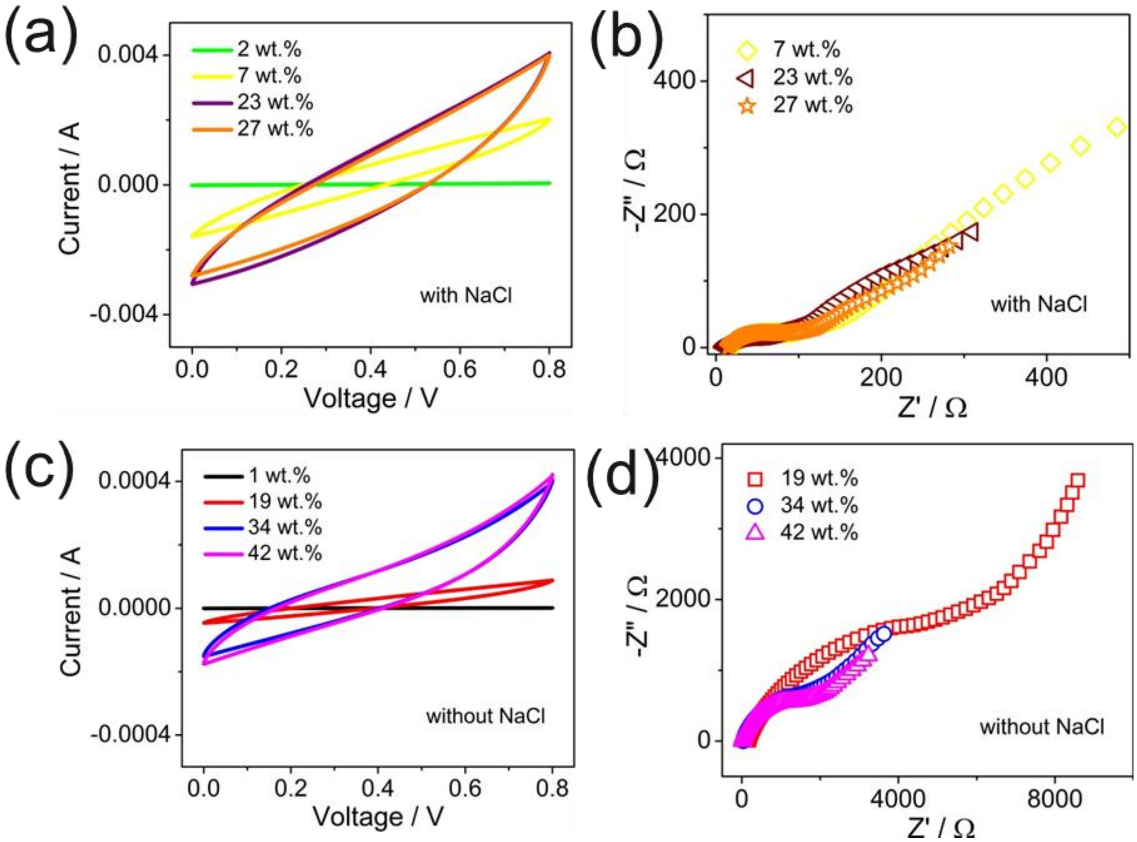

Figure S8. CV profiles (a, c) and Nyquist plots (b, d) of the miwPU polyelectrolyte with various concentrations of water in the film with and without NaCl.

No matter there is NaCl or not, CV curves are remarkably different at various water contents. They get larger with increased water content in the miwPU polyelectrolyte film. The enhanced capacitances can be attributed to high ion mobility at high water contents and convenient ion transfer in the moisturized electrolyte/electrode interface. Electrochemical impedance spectroscopy measurements also reflect these results. The supercapacitors exhibit lower charge transfer resistance and overall impedance at higher water contents, in which ions move more easily and polymer chains are more sufficiently extended. These favor ion transportation in the electrolyte and at the electrolyte/electrode interface, thereby reducing the resistance and increasing the capacitance.

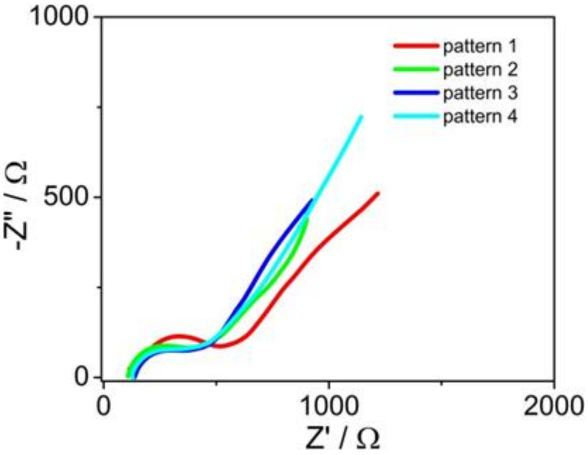

Figure S9. Nyquist plots of patterned miwPU artificial leather supercapacitors.

Electrochemical impedance spectra of these patterned miwPU artificial leather supercapacitor have no remarkable difference due to the fact that the patterns and colors do not affect the electrochemical dynamic processes in the supercapacitor.

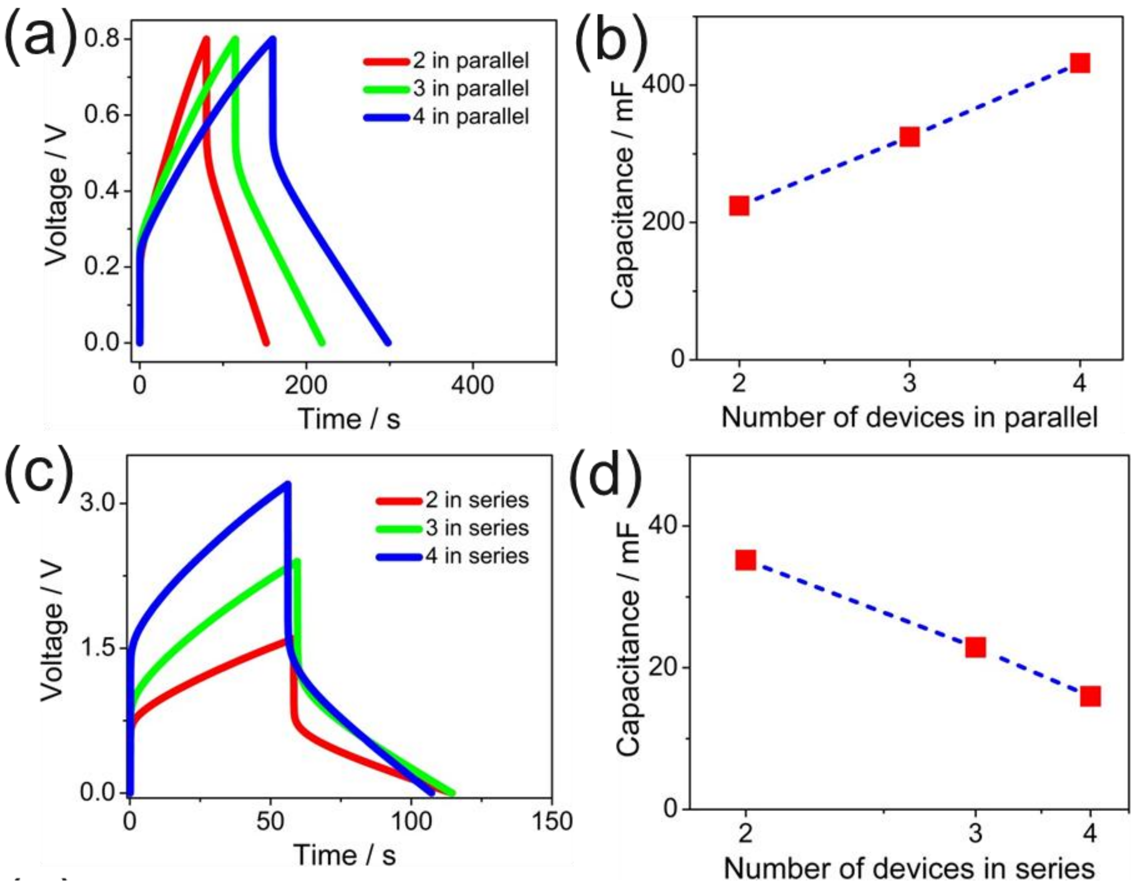

Figure S10. Electrochemical performances of multiple supercapacitors. (a) GCD curves of in-parallel assemblies. (b) Capacitances of in-parallel assemblies with repect to the number of devices. (c) GCD curves of in-series assemblies. (d) Capacitances of in-series assemblies with repect to the number of devices.

The charge/discharge time and thus the overall capacitance of the parallel assembly linearly increases with the number of supercapacitors, suggesting a good scalability. Similarly, compared with a single supercapacitor with an operating voltage of 0.8 V, the four-in-series assembly exhibits a four-fold wider charge/discharge voltage window (3.2 V) with similar discharge time. The overall capacitance of the in-series assembly linearly decreases with the reciprocal of the number of supercapacitors.

Table S1. Comparison of our PPy-based artificial leather supercapacitor with the reported PPy-based solid-state supercapacitor devices in terms of cycling stability, scan rate, capacitance and fashion demonstration.

Ref.	Electrode material	Cycling stability		Maximum scan rate recorded (V/s)	Capacitance (F/g)	Fashion demonstration
		Capacitance retention (%)	Cycle number			
This study	PPy@CNT	80	2500	1	105	Fluorescent, and pattern transferrable
S4	PPy	83.6<br>74.4	2000	0.1	51<br>49	
S5	PPy-nanoporous gold	78	900	1	250	
S6	PPy-PANI	60	4000	0.05	25	
S7	PPy-reduced graphene oxide	75	2000	0.1	37	
S8	PPy	78	1000	0.1		
S9	PPy-carbon fiber paper	93	2000	0.1	82.4	
S10	PPy-MnO_2	86.7	1000	0.2		

Supplementary Methods

Raman spectra were obtained by PERKINELMER Raman Station 400F Spectrometer with an excitation wavelength of 785 nm. Tensile strength of miwPU polyelectrolyte samples was measured by a mechanical testing system (Zwick-Roell Z005, Ulm, Germany) at a strain rate of 20 mm min^{-1}. The microstructure and morphology of electrodes were characterized by scanning electron microscope (SEM) (JEOL JSM-6335F) with an acceleration voltage of 5 kV. Electrochemical impedance spectra (EIS) were measured at frequencies ranging from 0.01 Hz to 100000 Hz with a potential amplitude of 5 mV.

Supplementary References

S1. Meera, K. M. S., Sankar, R. M., Paul, J., Jaisankara, S. N. & Mandal, A. B. The Influence of Applied Silica Nanoparticles on a Bio-renewable Castor Oil based Polyurethane Nanocomposite and Its Physicochemical Properties. *Phys. Chem. Chem. Phys.* **2014**, *16*, 9276-9288.

S2. Xia, H., Wang, Y., Lin, J. & Lu, L. Hydrothermal Synthesis of MnO_2/CNT Nanocomposite with a CNT Core/Porous MnO_2 Sheath Hierarchy Architecture for Supercapacitors. *Nanoscale Res. Lett.* **2012**, *7*, 1-10.

S3. Wang, J., Xu, Y. L., Yan, F., Zhu, J. B. & Wang, J. P. Template-Free Prepared Micro/Nanostructured Polypyrrole with Ultrafast Charging/Discharging Rate and Long Cycle Life. *J. Power Sources* **2011**, *196*, 2373-2379.

S4. C. Zhao, C. Y. Wang, Z. L. Yue, K. W. Shu, G. G. Wallace, ACS Appl. Mater. Interfaces 5 (2013) 9008–9014.

S5. F. H. Meng, Y. Ding, Adv. Mater. 23 (2011) 4098–4102.

S6. A. Clemente, S. Panero, E. Spila, B. Scrosati, Solid State Ion. 85 (1996) 273–277.

S7. W. H. Khoh, J. D. Hong, Colloids Surf. A 456 (2014) 26–34.

S8. C. Zhao, C. Y. Wang, R. Gorkin, S. Beirne, K. W. Shu, G. G. Wallace, Electrochem. Commun. 41 (2014) 20–23.

S9. C. Y. Yang, J. L. Shen, C. Y. Wang, H. J. Fei, H. Bao, G. C. Wang, J. Mater. Chem. A 2 (2014) 1458–1464.

S10. J. Y. Tao, N. S. Liu,W. Z. Ma, L. W. Ding, L. Y. Li, J. Su, Y. H. Gao, Sci. Rep. 3 (2013) 1–7.
